# Do Baseline Executive Functions Mediate Prospective Memory Performance under a Moderate Dose of Alcohol?

**DOI:** 10.3389/fpsyg.2016.01325

**Published:** 2016-08-31

**Authors:** James H. Smith-Spark, Antony C. Moss, Kyle R. Dyer

**Affiliations:** ^1^Division of Psychology, School of Applied Sciences, London South Bank UniversityLondon, UK; ^2^Institute of Psychiatry, Psychology and Neuroscience, King’s College LondonLondon, UK

**Keywords:** alcohol drinking, prospective memory, executive functions, memory for intentions test, updating, inhibition (psychology), set shifting, verbal fluency

## Abstract

Prospective memory (PM) is memory for delayed intentions. While deleterious effects of acute doses of alcohol on PM have been documented previously using between-subjects comparisons, the current study adopted a single blind placebo-controlled within-subjects design to explore whether the extent to which alcohol-related impairments in PM are mediated by executive functions (EFs). To this end, 52 male social drinkers with no history of substance-related treatment were tested using two parallel versions of a clinical measure of PM (the Memory for Intentions Test; Raskin et al., 2010), and a battery of EF measures. Testing took place on two occasions, with the order of administration of the alcohol and placebo conditions being fully counterbalanced. Overall, PM was worse under alcohol and participants showed deficits on five of the six subscales making up the clinical test. Hierarchical multiple regression analyses demonstrated that EFs did not predict PM performance decrements overall but did predict performance when time cues were presented and when verbal responses were required. Phonemic fluency was the strongest of the EF predictors; a greater capacity to gain controlled access to information in long-term memory predicted a smaller difference between placebo- and alcohol-related performance on both the time cue and verbal response scales. PM is crucial to compliance with, and response to, both therapy programs and alcohol harm prevention campaigns. The results indicate that individual differences in cognitive function need to be taken into account when designing such interventions in order to increase their effectiveness.

## Introduction

The adverse effects of alcohol upon cognitive function are well-documented. A consistent finding in the literature is that alcohol tends to impair higher-order, controlled cognitive processes selectively, whilst leaving automatic processes intact (e.g., Casbon et al., 2003; Moss and Albery, 2009). Executive functioning (EF) describes a range of high-level cognitive functions such as problem solving, planning, inhibiting automatic behavior in favor of more novel task-appropriate responses, self-monitoring performance, (e.g., Pennington and Ozonoff, 1996; Rabbitt, 1997; Stuss and Benson, 1997; Miyake et al., 2000; Andrés, 2003; Fisk and Sharp, 2004; Miyake and Friedman, 2012; Diamond, 2013). The current study focused on four distinct EFs, namely inhibition, set shifting, updating, and verbal fluency. Inhibition relates to the ability to withhold an automatic or habitual response in favor of more novel, task-appropriate behavior (e.g., Diamond, 2013). Set shifting refers to the ability to move flexibly between different cognitive operations or representational sets (e.g., Monsell, 2003). Updating reflects the ability to update information held in working memory in the light of new information (e.g., Miyake et al., 2000). Verbal fluency provides an index of the ability to gain controlled, flexible access to verbal information stored in long-term memory (e.g., Fisk and Sharp, 2004). Alcohol has been shown to have deleterious effects on EF (e.g., Peterson et al., 1990; Giancola and Moss, 1998; Blume et al., 2000; Lyvers and Tobias-Webb, 2010). As a result, its effects may also extend to having an impact on abilities which are argued to rely upon EFs. The research reported in the current paper was conducted in order to explore the potential mediating role of EFs in reducing alcohol-related declines in one such area, namely that of prospective memory (PM; memory for delayed intentions; e.g., Winograd, 1988), in adult male social drinkers.

An individual uses PM when “remembering to remember” (Mäntylä, 1994). There are two key cognitive components to any PM task (e.g., Einstein and McDaniel, 1990, 1996). The prospective (or planning) component is concerned with remembering to perform the intended action at the appropriate point in the future. The retrospective component involves remembering the nature of the intended action itself. There are two main types of demand placed on the PM system. Event-based PM demands require an intended action to be called to mind in response to the occurrence of a particular event in the environment (e.g., needing to pass a message on to a colleague and seeing her in the corridor should act as the cue to pass on that message). With event-based PM, environmental cues should, thus, act to trigger the appropriate behavior at the appropriate point in the future. Time-based PM demands, on the other hand, require an intended action to be performed at a certain time in the future (e.g., telephoning a colleague in 30 min) and time-based PM usually operates in the absence of salient external cues to support remembering. Instead, self-initiated mental processes (such as free recall) are relied upon to guide remembering to perform the action in the future (e.g., Einstein et al., 1995). Since self-generated strategies for remembering are required, time-based PM has been argued to involve EFs to a greater extent than event-based PM (e.g., McDaniel and Einstein, 2000; Martin et al., 2003; although see Huang et al., 2014).

Prospective memory has been identified as a potential contributor to relapse in problem drinkers, with Leitz et al. (2009) arguing that relapse could be viewed in part as a failure of PM. As Griffiths et al. (2012) have noted, learning-based therapies in alcohol treatment require an individual to carry out an intention (i.e., to abstain from drinking) at a point removed in time and location from the therapy session in which the intention was formed. With alcohol treatment in mind, it is therefore of concern that alcohol itself has been found to have both chronic and acute effects on the very memory system needed to remember such intentions (e.g., Leitz et al., 2009; Heffernan et al., 2010). More generally, the study of PM failures under alcohol is important to health behavior since many interventions targeted at non-dependent drinkers rely, to some extent, on PM. For example, remembering the intention to order food from a bar menu while drinking or remembering to leave a bar at a specific time are instances of event-based PM and time-based PM, respectively. Whilst there are many factors which will determine drinking behaviors once an individual is intoxicated, PM failure may well be an important factor in leading to harmful consequences.

The evidence for the deleterious effects of alcohol on PM comes predominantly from self-report studies which have documented its chronic effects on teenaged binge-drinkers and heavy-drinking university students (see Heffernan, 2008, for a review; see also Heffernan et al., 2010). These studies used self-report questionnaires to assess the relative frequency of different types of PM failure in everyday life. The results generally indicated the long-term harm of heavy or excess alcohol use on PM across a range of different everyday PM demands, even after controlling for differences in the use of strategies to facilitate successful PM. Experimental evidence demonstrating the adverse long-term effects of heavy drinking or alcohol dependence on PM has also been reported (Heffernan et al., 2010; Arana et al., 2011; Griffiths et al., 2012; Winward et al., 2014).

Further to this, and of greater relevance to the current study, there is also a small literature reporting the acute effects of alcohol on PM in non-problem drinkers (Leitz et al., 2009; Paraskevaides et al., 2010; Montgomery et al., 2011). The studies have used between-subjects designs to find evidence of alcohol-induced PM deficits across different tasks.

Leitz et al. (2009) administered alcohol at 0.6 g/kg blood alcohol content to social drinkers (consuming 2–14 units per week on average) in a double-blind matched placebo between-subjects design. They found significantly poorer PM performance in the alcohol group across measures of both regular and irregular PM and both time-based PM and event-based PM. On the basis of these findings, the authors concluded that four to five units of alcohol were sufficient to affect PM performance in daily life. Further to this, Leitz et al. (2009) found that participants’ performance on the Tower of London task, a measure of EF, was unimpaired by alcohol, leading them to argue that alcohol affects the retrospective component of PM rather than the planning component which calls upon EFs.

Again using the same PM task as Leitz et al. (2009); Paraskevaides et al. (2010) administered 0.6 g/kg Blood–Alcohol Content to undergraduate social drinkers whose average weekly alcohol consumption was 2–14 units. Event-based PM was found to be impaired by alcohol but not time-based PM. Paraskevaides et al. (2010) argue that event-based PM has a larger episodic memory component than time-based PM, with intentions being associated with their context, and that alcohol has an adverse effect on source memory but not EFs. However, the authors made clear that alcohol’s effects on PM cannot be attributed solely to its impact on the retrospective component since it had deleterious effects on regularly enacted PM tasks (and these place the smallest demands on retrospective memory). Paraskevaides et al. (2010) argued that alcohol may influence the quality of the episodic content of the prospective component instead of on remembering what the intention was, impairing the “development of a rich visual-spatial context around the plan” (p. 307). The failure to replicate Leitz et al.’s (2009) findings on time-based PM was explained in terms of a reduced number of trials and time monitoring behavior drawing on environmental cues rather than being purely self-initiated.

Montgomery et al. (2011) administered a 0.4 g/kg dose of alcohol to male and female university students who had consumed at least 10 UK units of alcohol in the previous week and had consumed four UK units at one sitting in the past month. PM performance was measured using the Jansari–Agnew–Akesson–Murphy task (JAAM; Jansari et al., 2004). Montgomery et al. (2011) found that there were deleterious effects of alcohol on the planning, prioritization, creativity, and adaptability EF subscales of the JAAM. Of more direct relevance to the present study, they also found lower event-based PM and time-based PM, but not the action-based PM subscale (where a stimulus in the current task in which they were engaged was meant to trigger PM performance). Furthermore, the planning EF subscale was found to be a significant predictor of both event-based PM and time-based PM and was close to significance for action-based PM.

Research has thus investigated the effects of alcohol on PM, finding that it has objectively measured deleterious effects both chronically and acutely and in both heavy/problem drinkers and non-problem social drinkers (e.g., Leitz et al., 2009; Heffernan and O’Neill, 2012). The doses involved in such studies are relatively low, often being below the legal limit for driving a vehicle in many jurisdictions. Moreover, the effects of alcohol on EFs are well-documented (e.g., Hildebrandt et al., 2004; Field et al., 2010; Montgomery et al., 2012), such that alcohol is known to selectively impair EF and other higher-order cognitive processes, leaving automatic processes intact (Fillmore et al., 1999; Moss and Albery, 2009). This is pertinent to research exploring the effects of alcohol on PM function, to the extent that different PM functions are likely to be underpinned by more, or less, automatic processes.

As stated previously, EFs have been argued to play a role in PM (e.g., Cockburn, 1995; Burgess and Shallice, 1997; Schnitzspahn et al., 2013). For example, Van den Berg et al. (2004) have argued that EFs are needed in order to break out from an ongoing task in order to perform the PM intention. Martin et al. (2003) have proposed that EFs are involved in forming and executing intentions but play less of a role in the retaining of intentions. Several EFs, in particular, have been found to be linked to PM performance in adults, namely inhibition, set shifting, and working memory. Inhibition has been found to be positively associated with time-based PM (Gonneaud et al., 2011). Set shifting, too, has been reported to be positively related to event-based PM (Bisiacchi et al., 2009; Gonneaud et al., 2011). Working memory (argued to be conceptually very similar to updating; Chein et al., 2011) has also been found to be a positive predictor of PM performance (e.g., Marsh and Hicks, 1998; Smith et al., 2011), especially under high cognitive load (Basso et al., 2010). Set against this evidence, however, Altgassen et al. (2014) found no predictive relationships between measures of updating, inhibition, and switching and PM in their sample of young adults.

The frontal lobes are typically associated with EFs (e.g., Rabbitt, 1997) and there is growing evidence indicating that they are also involved in PM function (e.g., McDaniel et al., 1999; McFarland and Glisky, 2009). Neuropsychological work has also identified a role for the frontal lobes in PM with inhibitory processes being identified by Cockburn (1995) as necessary in breaking out from ongoing activity to perform a delayed intention. Anterior regions of the prefrontal cortex (PFC) have been found to be more greatly activated when a delayed intention is added to the performance of an ongoing task (e.g., Okuda et al., 1998), with Brodmann’s Area (BA) 10 being particularly associated with PM (e.g., Burgess et al., 2003; although see Kalpouzos et al., 2010, who found no activation of BA10 on a naturalistic PM task). McDaniel et al. (2015) identify two pathways involved in PM, with reflexive associative processes being mediated primarily by the hippocampus and medial temporal lobes, whilst tasks requiring monitoring for PM cues lead to frontoparietal activation (e.g., Cona et al., 2015). Okuda et al. (2011) argue for distinct contributions of different PFC aspects. From their perspective, lateral PFC is involved in the maintenance of delayed intentions during ongoing task performance (c.f. Burgess et al., 2001; Simons et al., 2006), whilst medial PFC is engaged in the detection of PM targets, with there being deactivation of this area subsequent to the identification of, and response to, a target (c.f. Burgess et al., 2003; Simons et al., 2006). Okuda et al. (2011) propose a “gateway” hypothesis in which medial PFC coordinates attention reflexively in response to environmental input whilst lateral PFC is required for the controlled disengagement of attention from stimuli and the reorientation of attention to internal representations of intended actions.

Executive functions have, thus, been linked to PM performance both at both cognitive and neuropsychological levels but evidence is lacking concerning their potential mediating role in PM under the effects of alcohol. Individual differences in these EFs may mediate the effects of alcohol on PM, such that pre-existing deficits in one or more of these functions may predispose an individual toward greater PM failure under alcohol. Stated differently, stronger EF abilities may serve as an insulating factor against PM failures under alcohol. Whilst some studies have assessed PM and EF in the same sample (e.g., Heffernan et al., 2004; Paraskevaides et al., 2010), the vast majority have not directly assessed the effects of alcohol on the EFs which have been reported to facilitate PM (e.g., Bisiacchi et al., 2009; Gonneaud et al., 2011), so it is not possible to determine the mechanism via which alcohol may impair PM function via EFs, in either acute or chronic cases. Arana et al. (2011) did test this link explicitly but did not find that their EF measure predicted either event-based PM or time-based PM performance in university students. Similarly, Griffiths et al. (2012) found no correlations between several EF measures and any of their PM indices in social drinkers (although they did find a negative correlation between the time taken to complete Part B of the Trail-Making Test and performance on regular event-based PM tasks in abstinent alcohol-dependent participants).

The current experiment was, therefore, conducted to understand the role of EFs in alcohol-related declines in PM using a single-blind placebo-controlled within-subjects design. Whilst the choice of different EFs which could, potentially, have been studied is quite large, resource limitations in terms of researcher time and the need to retain the goodwill of participants in not subjecting them to a lengthy battery of mentally taxing tasks, four distinct EFs were selected for study. Three of the measures chosen to assess EF drew on functions identified within the unity/diversity framework (Miyake et al., 2000; Miyake and Friedman, 2012), namely inhibition, updating, and set shifting. The literature reviewed earlier in this section had already highlighted links between these EFs and PM (e.g., Einstein et al., 1995; Bisiacchi et al., 2009; Gonneaud et al., 2011), thus providing a good starting point for determining how alcohol might mediate their influence. Verbal fluency, a further well-recognized EF (e.g., Fisk and Sharp, 2004), was also assessed in order to explore how the ability to gain controlled and flexible access of information in long-term memory might predict PM performance under alcohol. Given the recorded effects of alcohol on retrospective memory (e.g., Curran and Hildebrandt, 1999; Söderlund et al., 2005), the influence of baseline verbal fluency ability seemed to have utility to explore. Two measures were taken, one assessing phonemic fluency (the ability to generate words with beginning with a particular letter) and the other measuring semantic fluency (the ability to produce words belonging to a certain semantic category). Of the two, phonemic fluency is argued to have more novel task requirements since it is a more usual activity to generate semantic associates and, as a result, cognitive schemata already exist to produce semantically related items (Troyer et al., 1997; Shao et al., 2014).

The Memory for Intentions Test (MIST; Raskin et al., 2010) was used to assess PM performance. There are two parallel versions of the MIST, making it ideal for within-subjects research on PM. Consistent with previous between-subjects work, in which the adverse acute effects of alcohol on PM have been highlighted (e.g., Leitz et al., 2009; Paraskevaides et al., 2010; Montgomery et al., 2011), lowered performance was expected under alcohol. This research also suggested that differences between different types of PM might be found (particularly between event-based PM and time-based PM) and that retrospective memory for PM instructions might also be deleteriously affected (e.g., Curran and Hildebrandt, 1999; Söderlund et al., 2005). In order to determine the impact of alcohol on PM relative to performance under the placebo, the difference between scores under the two conditions was calculated for each participant. Scores on the EF measures were entered into hierarchical regressions to determine whether they predicted the extent of the difference in shown by participants in their PM scores between the alcohol and placebo conditions.

## Materials and Methods

### Participants

Fifty-two male university students, native English speakers aged between 18 and 36 years (mean = 25 years, *SD* = 5), were paid a small honorarium or received course credit for participation in the study. They were all non-alcohol dependent social drinkers with a mean social drinking experience of 8 years (*SD* = 5). Due to the sample size involved in the study, the decision to restrict participants to males only was taken to reduce variability in physiological responses to alcohol. Before testing, the participants were asked to complete the Alcohol Use Disorders Identification Test (AUDIT; Babor et al., 2001). In order to be eligible to participate, the participants were required to score a minimum of one and a maximum of 20 on the first three questions of the AUDIT. A score of 21 points or more on these AUDIT questions suggests alcohol dependence. The mean AUDIT score of the sample was 9.50 (*SD* = 4.42). Further to this, the participants were required to be free of physical health complications (e.g., heart disease or gastrointestinal illness) if the ingestion of alcohol would put them at risk of secondary effects due to their current medication. Finally, individuals reporting a history of being treated for substance misuse problems were excluded from the study.

### Materials and Tasks

The MIST (Raskin et al., 2010) consisted of eight PM tasks embedded within a 30-min ongoing task. Parallel versions of the MIST (MIST-A and MIST-B; Raskin et al., 2010) were administered. The MIST-A was always presented in Session 1 and the MIST-B was always administered in Session 2. For both versions, the participants were instructed to make a particular response at the appropriate time (e.g., “In 15 min, tell me to check my mail.”) or in response to the appropriate event (e.g., “When I hand you a red pen, sign your name on your paper.”) whilst, in the meantime, being engaged in solving a word search puzzle. Each MIST task was scored from zero to two; a score of two indicated that the task was performed correctly, a score of one that it was performed partially correctly, and a score of zero that it was not performed at all. Six scales (2-min, 15-min, time-based, event-based, verbal, and action) were derived from the MIST, each calculated from responses to four of the eight PM tasks presented. The 2- and 15-min scales referred to the delay between being given the PM instruction and having the opportunity to act upon it. The time- and event-based scales related to the type of PM entailed in performing the task (e.g., whether a particular response was required after a certain amount of time had elapsed or when a particular event occurred, such as being passed a pen by the experimenter). Different types of PM response were required of the participants and these were reflected in the final two scales, namely verbal (e.g., telling the experimenter to check his or her mail) and action (e.g., the participant signing his or her name). Scores on each scale could vary from zero to eight, resulting in a maximum total score of 48.

After finishing the MIST, the participants had an eight-item multiple choice retrospective recognition questionnaire read out to them by the researcher. One question related to the content of each of the PM instructions given in that particular version of the test. The participants were asked to select the correct PM instruction from a choice of three options.

Three lists of 30 double-digit numbers were presented in the Plus–Minus task (Jersild, 1927). The participants were instructed to work as quickly and as accurately as they could to complete each list. In response to the first list (Add-3), the participants were asked to add three to each of the double-digit numbers presented. On the second list (Minus-3), the participants were requested to subtract three from each double-digit number. In the third and final list (Plus–Minus), the participants alternated between adding three to and subtracting three from each double-digit number. The time taken to complete each list was recorded by the experimenter. A measure of switch cost was derived from the Plus–Minus task by calculating the mean time to complete the Plus-3 and Minus-3 trials and subtracting this mean time from the time taken to finish the Plus–Minus trial.

Two measures of verbal fluency were administered from the Delis–Kaplan Executive System (Delis et al., 2001). The Letter Fluency subscale required participants to name out loud as many words (excluding proper nouns) as they could in 1 min. Three separate trials were administered, requiring the generation of responses to the letters F, A, and S. The Category Fluency subscale consisted of two 1-min trials, one requiring the generation of animal names and the other demanding the production of boys’ names. The mean number of valid words generated was calculated for the Letter Fluency and the Semantic Fluency tasks to produce measures of phonemic and semantic fluency, respectively.

The Go/No Go task required participants to inhibit a pre-potent response when presented with a less frequently occurring stimulus. The task consisted of 200 trials. Two line drawings (of a kangaroo and a sheep), matched for visual complexity, were taken from the Snodgrass and Vanderwart (1980) picture set and used as stimuli in the Go/No Go task (Luria, 1966). The line drawing of the kangaroo served as the pre-potent (or habituated) stimulus and the line drawing of the sheep acted as the non-habituated stimulus. The participants were asked to press the “s” key on every trial on which the line drawing of the kangaroo was presented and instructed to refrain from making any keyboard response whenever the line drawing of the sheep appeared. The first 40 trials consisted entirely of presentations of the habituated stimulus and all thus required a key-press response. After building up this pre-potent response, the remaining 160 trials were made up of 140 habituated and 20 non-habituated stimuli and followed seamlessly on from the habituation phase. The non-habituated stimuli were presented semi-randomly, every three to six trials. For a similar ratio of habituated to non-habituated stimuli, see Smith-Spark et al. (2016). The number of inhibition trials on which the participant correctly prevented a motor response was recorded and expressed as a percent accuracy value.

The Automated Operation Span (Conway et al., 2005; Unsworth et al., 2005), a test of executive-loaded working memory, was used to assess updating. The participants were asked to solve a series of simple arithmetic problems whilst also remembering a sequence of letters. Initially, the participants completed separate practice sessions in which they attempted the two different components of the task. Firstly, they carried out a letter span task, requiring them to recall sequences of letters in serial order. Secondly, they practiced performing a series of simple mathematical operations. At the end of the mathematics practice phase, the program automatically calculated the mean length of time required to solve the mathematics problems. This mean duration plus 2.5 standard deviations was used to form the time limit for the mathematical operations in the experimental phase. After this time limit had been exceeded, a trial was marked as incorrect. It has been argued by Unsworth et al. (2005) that this process of individual titrations permits individual differences in the time taken to solve mathematical problems (i.e., processing speed) to be controlled and prevents participants from rehearsing the letters verbally when they should instead be solving the problems themselves. After this, the letter span and mathematics task requirements were combined to form a final practice phase. During this final practice (and during the experiment itself), the participants were shown a series of mathematical problems (e.g., 1*2). Each problem was followed by the visual presentation of a further number. The participant was instructed to indicate with a mouse click whether or not the number matched the answer to the preceding arithmetic problem. After the production of a true/false response, a letter was then presented onscreen for serial recall at the end of the trial. The number of arithmetic problems which were shown per trial varied between three and seven, with there being three trials at each set length. Two measures were derived from the Operation Span task. The first of these, Operation Span score, was the sum of all the sets of letters correctly recalled by the participant. The second, Operation Span total correct, was the number of letters recalled in the correct serial position, regardless of whether trial performance as a whole was correct.

Becks Bier 5% ABV and Becks Blue Alcohol-Free Beer were used, respectively, as the alcoholic and placebo drinks.

### Design

A single-blind placebo-controlled within-subjects design was employed. Testing was conducted on two occasions, 7–14 days apart. In all analyses of variance (ANOVAs) and multivariate analyses of variance (MANOVAs), order of administration (whether alcohol or placebo was administered in Session 1) was entered as a between-subjects factor.

As well as analyzing MIST total score and scores for each of the six scales, error type data were also investigated, assigning participants’ errors to the five categories identified by Raskin et al. (2010). The error types were no response (where the participant failed to make any PM response), loss of content (where the participant indicated that a PM response should be made but could not remember what to do), loss of time (where a PM response was made but its timing was wrong, being more than a minute earlier or later than the specified time for its execution), task sublimation (where an action response was substituted for a verbal response or vice versa, or where any previously given response was produced in place of the required response), and random error (where an incorrect response was made that did not fit any of the error types described above).

A difference score was calculated for total MIST score and the six MIST scale scores. In each case, this was done by subtracting a participant’s score in the alcohol condition from the participant’s corresponding score in the placebo condition. The difference scores, therefore, represented the extent to which performance improved or declined in the presence of alcohol. The difference scores so obtained were entered as dependent variables into hierarchical multiple regression analyses.

### Procedure

The research was granted full ethical approval from the University Research Ethics Committee at London South Bank University. All of the participants gave informed written consent to take part in accordance with the Declaration of Helsinki. At the start of the first testing session, the participants were asked to complete the AUDIT (Babor et al., 2001). Those that met the inclusion criteria were then weighed in order to determine how much alcohol should be administered in order for them to reach the target breath-alcohol concentration of 0.06 g/kg breath-alcohol content.

At the start of Session 1, a block randomization method was used to assign the participants to one of two condition orders in which they either ingested alcohol or received the alcohol-free placebo in the first session. After being assigned to one or other condition order (and before they were asked to ingest a drink), the participants performed the EF tests in the following fixed order: Plus–Minus, Letter Fluency, Category Fluency, Go No/Go, and Operation Span.

After completing the EF tasks, the participants were given a beverage to ingest. In the alcohol condition, the volume required to bring the participant to the required breath-alcohol concentration (BrAC) of 0.06 g/kg was calculated using the weight of the participant recorded at the start of the session. This volume was then given to the participant to imbibe. The participants were instructed to consume all of the drink within 30 min of being given it but in no less than 20 min. The drinks were divided into four glasses, each of which contained equal amounts of fluid. After the beverage had been completely consumed, the experimenter breathalyzed the participant for the first time to determine whether or not they had reached the target BrAC of 0.06 g/kg. The participants were then breathalyzed at 15-min intervals until they reached the target BrAC. The same volume of alcohol-free beer and instructions were given to the participants in the placebo condition. The participants were also breathalyzed on several occasions in the placebo condition.

Once the target BrAC was reached (or, in the case of the placebo being administered, after a 35-min interval), the MIST-A was presented, with the Retrospective Recognition Questionnaire being administered once the MIST-A was completed.

In Session 2, the participants either ingested alcohol if they had received the placebo in Session 1 or received the placebo if they had consumed alcohol in the first session. The alcohol administration process was exactly the same as that performed in Session 1. The MIST-B was administered, including Version B of the Retrospective Recognition Questionnaire.

A full verbal debriefing followed the completion of the MIST-B.

## Results

Two participants were removed from the data set prior to statistical analysis (and are, therefore, not reported in the Method). One was considerably older than the remaining participants (being 44 years of age) and the other participant produced an AUDIT score of 21. The removal of these participants resulted in 25 participants receiving the placebo condition on the first time of testing and 27 receiving alcohol the first time that they were tested.

### Performance on the MIST

#### Overall Performance

When the order of administration was considered, performance was rather better overall for the group presented with the placebo (mean = 44.64, *SEM* = 0.77) than it was for the group presented with alcohol (mean = 42.56, *SEM* = 0.74) on the first time of testing. However, there was no significant effect of order of administration on MIST total score, *F*(1,50) = 3.84, *MSE* = 29.417, *p* = 0.056.

On average, PM was more accurate under the placebo condition (mean = 45.67, *SEM* = 0.52) than under the alcohol condition (mean = 42.56, *SEM* = 0.76). A two-way mixed-measures analysis of variance indicated that there was a highly significant effect of administration condition on MIST total score, *F*(1,50) = 31.81, *MSE* = 14.065, *p* < 0.001, $\eta_{p}^{2}$ = 0.389.

There was no significant order of administration × administration condition interaction, *F*(1,50) < 1, *MSE* = 14.065, *p* = 0.923.

#### Performance on the Individual MIST Scales

On average, performance was worse under alcohol on all six MIST scales. A two-way mixed-measures MANOVA indicated that there was no significant effect of order of administration on scale scores, Wilks’ Λ = 0.896, *F*(4,47) = 1.36, *p* = 0.263. The two-way MANOVA showed that there was a significant multivariate effect of administration condition on the MIST scale scores, Wilks’ Λ = 0.602, *F*(4,47) = 7.77, *p* < 0.001, $\eta_{p}^{2}$ = 0.398. **Table 1** shows the descriptive statistics under the placebo and alcohol conditions and the univariate *F* test results for each of the six MIST subscales. The univariate *F* tests indicated that the presence of alcohol resulted in significantly lower scores on five of the six MIST scales (all at *p* ≤ 0.007). Event-based performance (*p* = 0.017) was not significantly affected by alcohol once a Bonferroni-corrected α-level of 0.008 was applied.

**Table 1:** Descriptive and univariate *F* test results for the six MIST scale scores under the two administration conditions.

	Placebo mean score (*SD*)	Alcohol mean score (*SD*)	*F*(1,50)	*MSE*	*p*	$\eta_{p}^{2}$
Two-minute delay	7.87 (0.44)	7.52 (0.85)	7.79	0.397	0.007	0.135
Fifteen-minute delay	7.35 (1.06)	6.31 (1.57)	27.47	1.019	<0.001	0.355
Event cue	7.81 (0.60)	7.42 (1.07)	5.99	0.643	0.018	0.107
Time cue	7.40 (0.98)	6.40 (1.32)	24.91	1.039	<0.001	0.333
Verbal response	7.62 (0.80)	7.02 (0.98)	15.78	0.585	<0.001	0.240
Action response	7.60 (0.80)	6.81 (1.40)	17.01	0.946	<0.001	0.255


*Bonferroni-corrected α-level = 0.008.*

There was no significant interaction between order of administration and administration condition, Wilks’ Λ = 0.994, *F*(4,47) < 1, *p* < 0.991.

#### Error Type Data

A further two-way mixed-measures MANOVA was performed on the error type data. There was no significant effect of order of administration on error production, Wilks’ Λ = 0.824, *F*(5,46) = 1.97, *p* = 0.101. However, there was a significant multivariate effect of administration condition on the type of errors produced in response to the MIST, Wilks’ Λ = 0.527, *F*(5,46) = 8.26, *p* < 0.001, $\eta_{p}^{2}$ = 0.473. The descriptive statistics and univariate test statistics for each error type under the two administration conditions are displayed in **Table 2.** The univariate *F* tests indicated that alcohol led to a higher frequency of error on two error types. The participants failed to make a PM response significantly more often under the alcohol condition. Significantly more loss of content errors were also made under the alcohol condition than under the placebo condition. There were no significant differences between the conditions on loss of time errors, substitution errors, or random errors.

**Table 2:** Descriptive and univariate *F* test statistics for the different MIST error types under the two administration conditions.

Error type	Placebo mean score (*SD*)	Alcohol mean score (*SD*)	*F*(1,50)	*MSE*	*p*	$\eta_{p}^{2}$
No response	0.08 (0.27)	0.48 (0.73)	20.56	0.204	<0.001	0.291
Loss of content	0.23 (0.43)	0.58 (0.70)	13.13	0.238	0.001	0.208
Loss of time	0.23 (0.47)	0.31 (0.51)	1.03	0.156	0.316	0.020
Substitution	0.08 (0.27)	0.15 (0.46)	1.09	0.136	0.302	0.021
Random	0.02 (0.14)	0 (-)	0.93	0.010	0.341	0.018


*Bonferroni-corrected α-level = 0.010.*

#### Retrospective Recognition Questionnaire

On average, the retrospective recognition score (mean = 7.57, *SEM* = 0.08) for the participants given alcohol in Session 1 was slightly lower than that of the participants given the placebo in the first session (mean = 7.84, *SEM* = 0.08). A two-way mixed measures analysis of variance indicated that there was a significant effect of order of administration on retrospective recognition, *F*(1,50) = 5.27, *MSE* = 0.348, *p* = 0.026, $\eta_{p}^{2}$ = 0.095.

The participants recognized significantly more instructions correctly under the placebo condition (mean = 7.80, *SEM* = 0.066) than under the alcohol condition (mean = 7.62, *SEM* = 0.078), *F*(1,50) = 4.16, *MSE* = 0.192, *p* = 0.047, $\eta_{p}^{2}$ = 0.077.

There was no significant administration order x adminis tration condition interaction, *F*(1,50) < 1, *MSE* = 0.192, *p* = 0.457.

### Performance on the EF Measures

The mean scores on the EF measures are displayed in **Table 3.**

**Table 3:** Descriptive statistics for the EF measures.

Measure	Mean score	*SD*	Minimum–Maximum
Plus–minus switch cost (s)	10.56	10.98	-18–45.50
Go/No Go accuracy (%)	92.55	7.67	65–100
Operation span score	43.71	14.62	7–71
Letter fluency mean number of items generated (over three trials)	18.00	4.17	9.33–27.33
Category fluency mean number of items generated (over two trials)	23.03	4.41	12.00–34.50


#### Executive Functions as Predictors of Alcohol-Induced PM Deficits

A hierarchical multiple regression analysis was run on the overall MIST difference scores, with order of alcohol administration entered in Block 1 and the EF measures entered in Block 2. The final model did not significantly predict overall MIST difference score, *R* = 0.374, *F*(6,45) = 1.22, *p* = 0.315. The *R*^2^ change and standardized β-values are shown in **Table 4** for all analyses conducted in this subsection.

**Table 4:** Summaries of the hierarchical multiple regression analyses conducted on the MIST difference scores.

MIST difference measure	Total *R*^2^	Δ*R*^2^ Block 2	β Order of alcohol administration	β Phonemic fluency score	β Semantic fluency score	β Plus–minus switching score	β Go/No Go accuracy	β Operation span score
Overall	0.140	0.140	-0.009	-0.303	0.043	0.140	-0.161	-0.014
Two-minute delay	0.094	0.093	-0.017	-0.260	0.228	0.169	-0.022	-0.044
Fifteen-minute delay	0.119	0.119	0.0003	-0.213	-0.089	0.067	-0.185	0.011
Event cue	0.042	0.042	0.026	-0.039	0.072	-0.041	0.168	0.097
Time cue	0.327^∗∗^	0.327^∗∗^	-0.031	-0.341^∗^	-0.003	0.203	-0.329^∗^	-0.093
Verbal response	0.254^∗^	0.254^∗^	-0.060	-0.376^∗^	0.070	0.332^∗^	-0.100	-0.026
Action response	0.031	0.030	0.036	-0.094	0.001	-0.082	-0.128	0.003


*For each regression, order of alcohol administration (0 = Placebo in Session 1, 1 = alcohol in Session 1) was entered in Block 1 and the EF measures were entered in Block 2. For Block 2, information is provided on the total variance accounted for by the model (total *R*^2^), change in *R*^2^ (Δ*R*^2^), and the standardized β-values for the predictor variables. ^∗^*p* < 0.05; ^∗∗^*p* < 0.01.*

Further hierarchical multiple regression analyses were run on the difference scores for each of the six MIST scales, with the order of alcohol administration entered in Block 1 and the five EF measures again entered in Block 2 as predictors into each model. In all cases, order of alcohol administration was not a significant predictor in Block 1 nor in the final model.

The EF measures were found to significantly predict the alcohol-related decline in time-based performance, *R* = 0.572, *F*(6,45) = 3.65, *p* = 0.005, with Go/No Go accuracy and Letter Fluency score being the only significant predictors in the final model. A stronger ability to inhibit responses on the Go/No Go task was associated with a smaller decline in PM performance under alcohol. Similarly, the ability to generate a greater number of words beginning with a particular letter was also associated with a smaller alcohol-related decline.

The alcohol-related difference on tasks requiring a verbal response was also significantly predicted by the EF measures, *R* = 0.504, *F*(6,45) = 2.56, *p* = 0.032, with Letter Fluency score and Plus–Minus score as the only significant predictors in the final model. The ability to shift flexibly between cognitive operations, indicated by a smaller cost of shifting on the Plus–Minus task, was associated with a smaller difference score. Again, there was also a negative association between phonemic fluency and difference score.

The EF measures did not significantly predict difference scores on the 2-min delay, *R* = 0.306, *F*(6,45) < 1, *p* = 0.592, 15-min delay, *R* = 0.346, *F*(6,45) = 1.02, *p* = 0.426, event cue, *R* = 0.206, *F*(6,45) < 1, *p* = 0.917, nor action-based response, *R* = 0.175, *F*(6,45) < 1, *p* = 0.962, scales.

## Discussion

The within-subjects design used in this experiment clearly demonstrated the deleterious effects of an acute administration of alcohol on PM. In terms of total score on Raskin et al.’s (2010) MIST, the participants scored lower under the alcohol condition than they did under the placebo. The effects of alcohol were very widespread, with scores on five of the six individual MIST scales being affected negatively by its presence. The presence of alcohol led to poorer performance in response to time cues, worse performance over both 2- and 15-min intervals, and affected task demands whether they required verbal or action-based responses. Only PM responses to event cues were not significantly affected by alcohol’s presence. These findings are consistent with evidence that alcohol impairs effortful cognitive processes – which have been argued to underpin time-based PM (McDaniel and Einstein, 2000; Martin et al., 2003) – while leaving more automatic processes – which have been argued, under certain circumstances, to underpin event-based PM (e.g., McDaniel and Einstein, 2000) – relatively intact (Moss and Albery, 2009).

Under alcohol, errors were more likely to take the form of entirely forgetting to perform the PM task (resulting in more No response errors) or else remembering that something needed to be done but failing to remember the actual contents of the intention (leading to more loss of content errors). The latter type of error may well be related to the effect of alcohol on retrospective memory (e.g., Curran and Hildebrandt, 1999; Söderlund et al., 2005), impairing the encoding, storage, or access of task-relevant information in memory. In support of this argument, alcohol was also found to have an adverse effect on participants’ ability to recognize PM instructions correctly after testing. Accuracy on the retrospective recognition questionnaire was significantly lower when participants had consumed alcohol. This result suggests problems with accessing verbal information in long-term memory in the presence of alcohol. This possible explanation for the findings is further reinforced by the predictive power of phonemic fluency (requiring the controlled access of verbal information in long-term memory; e.g., Fisk and Sharp, 2004) in determining the extent to which alcohol impaired PM performance. The contribution of EFs to PM under alcohol is considered in more depth later in this section.

The results of the present study are consistent with previous research in showing a deleterious acute effect of alcohol on PM function (Leitz et al., 2009; Paraskevaides et al., 2010; Montgomery et al., 2011) and, moreover, extend the range of PM tasks on which its effects have been documented. In the current study, the only measure of PM which seemed not to be impaired under alcohol was event-based PM. However, this result is not consistent with the findings of either Leitz et al. (2009) or Paraskevaides et al. (2010) who both found evidence for impaired event-based PM. It could be argued that the use of physical stimuli in the current study to prompt event-based PM responses (e.g., a pen being handed to the participant) served as more salient cues than those present in these previous studies, both of which used the same computerized task. In the current study, the increased cue salience would serve to facilitate automatic PM responses to a greater extent. Further to this, Montgomery et al. (2011) found no alcohol-related impairments when action-based PM responses were required. They defined this type of PM as one where participants are cued to respond by a stimulus within the task that they are currently engaged in completing. Given the nature of the event-based cues used in the present study, there is some overlap with Montgomery et al.’s (2011) “action-based responses” and this may explain why no differences were found on the event-based MIST scale.

Turning now to the question as to whether scores on EF measures can predict the extent to which alcohol leads to a decline in PM performance, the hierarchical multiple regression analyses indicated that predictive relationships did emerge on some, but not all, of the measures, after controlling for order effects at Step 1 of each model. Overall, there was no significant predictive relationship between EFs and PM with the predictor variables accounting for only 14% of the variance in total MIST score. However, significant relationships were found between some measures of EF and some individual MIST scales.

Consistent with the argument that time-based and self-initiated PM draw most heavily on EF resources (e.g., McDaniel and Einstein, 2000; Martin et al., 2003), performance on tasks with a time cue was predicted by inhibition and phonemic fluency. Higher phonemic fluency was associated with a smaller difference in PM performance between the placebo and alcohol conditions. A stronger ability to inhibit a pre-potent response was associated with smaller alcohol-related decline in performance. Inhibition, as measured by Go/No Go accuracy, was marginally the stronger predictor of the two. The final model accounted for 33% of the variance in the alcohol-related decline in time-based PM. This finding is consistent with research which has shown a relationship between alcohol use and EF performance. Specifically, Field et al. (2010) reviewed evidence which shows that the effects of alcohol on inhibitory control occur at doses which are less than those required to cause global cognitive impairment; that is to say, EF is particularly sensitive to alcohol. In addition, models of alcohol-related behavior change have argued that individual differences in EF moderate the effect of alcohol on behavior change, for example, in the context of alcohol-induced aggression (Giancola, 2000). This can be attributed to the differential impact of alcohol on controlled versus automatic cognitive processes (Tracy and Bates, 1999; Casbon et al., 2003; Moss and Albery, 2009, 2010; Field et al., 2010).

On the verbal response MIST scale, 25% of the variance in the alcohol-related decline in performance could be explained by the model in which phonemic fluency was the only significant predictor. Stronger phonemic fluency abilities, measured by Letter Fluency, were associated with a smaller alcohol-related decline in PM. A greater cost of switching on the Plus–Minus task was also associated with a greater alcohol-related drop in performance.

Whilst alcohol is known to impact on a range of effortful cognitive processes (e.g., Casbon et al., 2003; Moss and Albery, 2009), the present findings suggest that individual differences in pre-consumptive EF capacity have a mediating effect on the impact of alcohol on PM under certain conditions. Of the EFs tested, phonemic fluency (the ability to access information in long-term memory in a controlled and flexible manner; e.g., Fisk and Sharp, 2004), proved to have the most widespread predictive power in that stronger word generation abilities were associated with smaller PM performance declines on two MIST performance scales (namely time cues and verbal responses).

As noted previously, two further EFs were associated with the extent of PM decline under alcohol. Set shifting, the ability to move flexibly between cognitive sets or operations (e.g., Monsell, 2003), was a further predictor of alcohol-related declines when verbal responses were required. Finally, better inhibitory abilities (e.g., Diamond, 2013) were associated with a smaller drop in performance under alcohol when time cues were presented. The relationships highlighted by these latter two EFs suggest that alcohol affects different facets of moving between one task and another when required to perform a PM task. As argued by Cockburn (1995) and Burgess and Shallice (1997), a PM task requires an individual to break out from their ongoing activity at the appropriate point, inhibiting performance of the ongoing task in favor of the PM response (cf. Norman and Shallice, 1986). Set shifting, measuring the ability to move flexibly between one cognitive operation and another, could be considered another means by which behaviors are modulated according to changing priorities.

The present findings have shown a mediating role of EF on PM function. However, the measures of EF used were taken prior to the individual consuming alcohol. Future work to explore the mechanisms by which alcohol consumption leads to these impairments should include the measurement of EF abilities both pre- and post-alcohol consumption, in order to determine whether individual variations in the alcohol-related impairment of these processes themselves explains more of the variation in cognitive functions such as PM. Furthermore, the task impurity problem associated with measures of EF (e.g., Burgess, 1997; Miyake and Friedman, 2012) means that individual EF tasks tap cognitive processes which are incidental to executive processing. For example, in the present study, both the Plus–Minus task and the Operation Span task required mathematical processing, albeit demanding very simple, overlearned mathematical procedures (and, in addition, were both used by Miyake et al., 2000). The use of a battery of EF tasks to tap each EF domain is, therefore, recommended in preference to the single task per EF domain approach which was adopted in the present study due to time and resource constraints. These tasks should ideally call upon different processing domains or modalities. For example, inhibition abilities could be assessed using a Go/No Go task (Luria, 1966) together with an antisaccade task (e.g., Munoz and Everling, 2004) and/or a Stroop task (Stroop, 1935).

The results of the current study reinforce previous work (Leitz et al., 2009; Paraskevaides et al., 2010; Montgomery et al., 2011) in demonstrating that a moderate dose of alcohol can have detrimental effects on PM function. Given the centrality of PM to everyday life (McDaniel and Einstein, 2007), this effect warrants further investigation to understand the impact that alcohol-related PM failures might have on a range of health-related behaviors, amongst both problem and social drinking groups. More broadly, it is worth highlighting that the alcohol dose used in this and previous studies to demonstrate significant negative effects on PM function is equivalent to, and in many regions less than, the legal limit for driving a motor vehicle – a complex behavior which involves a range of PM-related actions which are more or less habitual in nature (e.g., remembering to take the correct turning on a road or check the fuel gauge). Further research specifically looking at the consequences of alcohol-related PM failure in such settings is therefore warranted to better understand the impact of alcohol on cognition in safety–critical environments where there is a need to remember to perform non-habitual or novel actions within, or instead of, a routine sequence of behaviors.

## Author Contributions

JS-S contributed significantly to the design of the study, the data analyses, and the interpretation of the findings, drafting and revising the manuscript, and agreed the submitted version. AM contributed significantly to the design of the study, data analysis and the interpretation of findings, drafting and revising the manuscript, and agreed the submitted paper. KD contributed significantly to the design of the study, revisions to the manuscript, and agreed the final version for submission. JS-S, AM, and KD all agree to be accountable for all aspects of the work.

## Conflict of Interest Statement

The authors declare that the research was conducted in the absence of any commercial or financial relationships that could be construed as a potential conflict of interest.

The reviewer GC and the handling Editor declared their shared affiliation, and the handling Editor states that the process nevertheless met the standards of a fair and objective review.
